# Association of imaging factors derived from convolutional neural network with visual outcomes in age-related macular degeneration and polypoidal choroidal vasculopathy

**DOI:** 10.1038/s41598-019-56420-z

**Published:** 2019-12-27

**Authors:** Hyungwoo Lee, Minsu Jang, Hyung Chan Kim, Hyewon Chung

**Affiliations:** Department of Ophthalmology, Konkuk University School of Medicine, Konkuk University Medical Center, Seoul, Republic of Korea

**Keywords:** Retinal diseases, Macular degeneration, Retinal diseases, Retinal diseases, Macular degeneration

## Abstract

We investigated the association of visual outcome in typical neovascular age-related macular degeneration (nAMD) and polypoidal choroidal vasculopathy (PCV) with or without pachychoroid with lesion areas on optical coherence tomography (OCT) quantified by convolutional neural network (CNN) analysis. Treatment-naïve 132 nAMD and 45 PCV eyes treated with ranibizumab or aflibercept for at least 12 months were retrospectively reviewed. Significant factors, including intraretinal fluid (IRF), subretinal fluid (SRF), pigment epithelial detachment (PED) and subretinal hyperreflective material (SHRM) area quantified by CNN at baseline and 12 months, were analyzed by logistic regression analyses for 3-line visual gain or maintenance of 20/30 Snellen vision. Visual gain at the final visit in nAMD was associated with a smaller SHRM at baseline (OR 0.167, P = 0.03), greater decrease in SRF and SHRM from baseline to month 12 (OR 1.564, P = 0.02; OR 12.877, P = 0.01, respectively). Visual gain in nAMD without pachychoroid was associated with a greater decrease in SRF and SHRM (OR 1.574, P = 0.03, OR 1.775, P = 0.04). No association was found in nAMD with pachychoroid and any type of PCV. Greater decrease in SRF and SHRM from baseline to month 12 was associated with favorable visual outcomes in nAMD without pachychoroid but not in nAMD with pachychoroid and PCV.

## Introduction

Neovascular age-related macular degeneration (nAMD) is the leading cause of blindness in adults over 50 years old^1^. Polypoidal choroidal vasculopathy (PCV) has been considered a variant form of nAMD that is more prevalent in Asian populations, but many characteristics specific to PCV have led to the concept that PCV might be a different disease entity from typical nAMD. Recent studies in the fields of genetics, imaging and clinical response have provided evidence of differences in pathophysiology between PCV and typical nAMD^2^. PCV is also considered a kind of pachychoroid spectrum disease, and one of the most common characteristics of this disease is outer choroidal thickening. Increased choroidal thickness in PCV is related to choroidal hyperpermeability, which is associated with a poor response to anti-vascular endothelial growth factor (anti-VEGF) treatment in PCV^3^. Moreover, “pachychoroid” were introduced to describe the choroidal environment including dilated outer choroidal vessels associated with attenuation of the overlying choriocapillaris regardless of choroidal thickness^4,5^. However, the differential pathophysiology and clinical course of PCV with or without pachychoroid are poorly understood.

Prognostic factors from multiple nAMD cohorts revealed that baseline visual acuity (VA) and optical coherence tomography (OCT) parameters, such as intraretinal fluid (IRF), subretinal fluid (SRF), pigment epithelial detachment (PED), or subretinal hyperreflective material (SHRM), were relevant to final VA and/or VA change^6–11^. Nonetheless, most studies including clinical trials did not analyze these prognostic factors according to nAMD subgroups, including PCV. In addition, the influence of pachychoroid on the visual prognosis of PCV remains unknown. Moreover, these studies used different OCT machines, not a single machine, or did not use spectral domain OCT (SD OCT)^12–14^. Therefore, analyses of detailed nAMD features or higher-dimensional information such as area of pathologic lesions might provide more accurate prognostic factors specific to the AMD subgroups.

The quantification of lesions on OCT by clinicians is a time-consuming task, and reliability among clinicians cannot be guaranteed. Deep learning is a swiftly advancing area of artificial intelligence, and it is now also applied in ophthalmology by automatically classifying images, segmenting lesions and even generating images based on trained data^15–17^. Recently, we developed and validated an automated segmentation algorithm based on a convolutional neural network (CNN), which can segment and calculate the area of IRF, SRF, PED and SHRM in SD OCT B-scan images^18^. Therefore, it became possible to precisely quantify the areas of lesions from a large number of patients in a very short time.

In the current study, we measured the area of IRF, SRF, PED and SHRM in typical nAMD and PCV at baseline and 12 months after the initial treatment with either ranibizumab or aflibercept using an automated segmentation algorithm. Additionally, we investigated the presence of pachychoroid in each case. Then, we analyzed the prognostic factors, including these anatomical parameters at baseline and 12 months, associated with visual improvement.

## Results

This study investigated 177 eyes of 169 patients, including 132 (74.6%) typical nAMD eyes and 45 (25.4%) PCV eyes. Detailed baseline characteristics of eyes with typical nAMD and PCV are depicted in Supplementary Table S1. The mean logMAR BCVA at all visits did not differ significantly between the two groups. The baseline SFCT of typical nAMD was thinner than that of PCV. Among the 4 segmented lesions, the baseline areas of SRF and PED were significantly smaller in typical nAMD than in PCV, while IRF and SHRM showed no differences at baseline (Supplementary Table S1).

**Figure 1:**
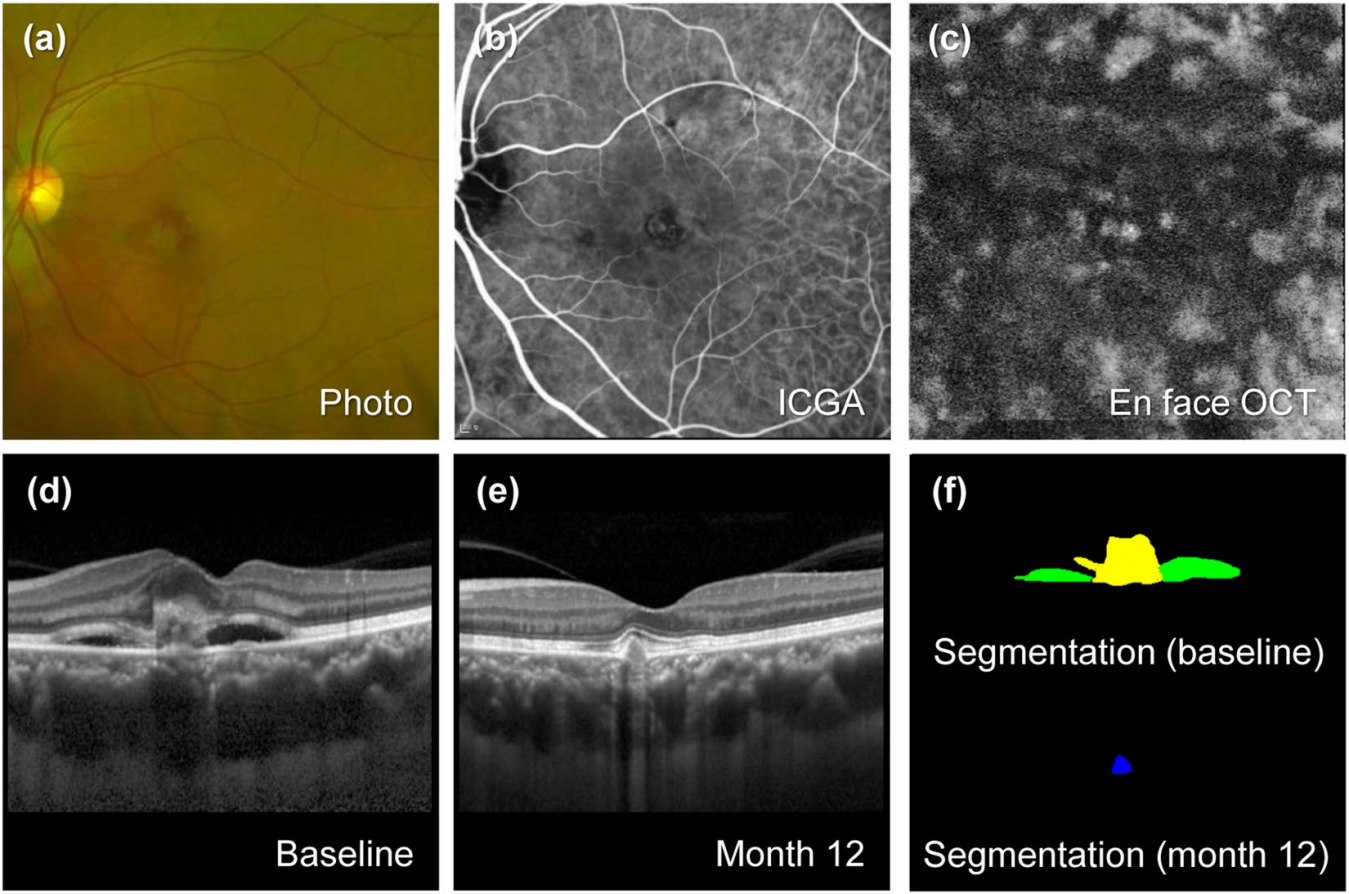
Representative case of typical neovascular age-related macular degeneration (nAMD) with pachychoroid. The patient is a 52-year-old man with a baseline visual acuity of 20/200. For 21.6 months of follow-up, the eye was treated with 5 intravitreal injections of aflibercept. (**a**) Baseline fundus photography shows subretinal hemorrhage accompanied by subretinal fluid. (**b**) Baseline indocyanine green angiography (ICGA) shows choroidal neovascularization (CNV) without polyps at the macula. (**c**) En face optical coherence tomography (En face OCT) scan at the final visit. Dilated choroidal vessels are distributed in the entire area. (**d**) Enhanced depth imaging optical coherence (EDI OCT) at baseline. The subfoveal choroidal thickness (SFCT) is increased as 509 µm. (**e**) EDI OCT scan after 12 months from baseline. Visual acuity at 12 months improved to 20/25. At final visit, visual acuity improved to 20/20. (**f**) Upper panel shows predicted lesion corresponding baseline OCT B-scan by a convolutional neural network (CNN) (upper). Subretinal fluid (SRF, green color), subretinal hyperreflective material (SHRM, yellow color) are noted. Each component could be quantified by converting the pixel counts into mm^2^, and the areas of SRF, SHRM are 0.146, 0.183 mm^2^, respectively. Lower panel shows pigment epithelial detachment (PED, blue color) area predicted from OCT B-scan at 12 months by CNN, and the area is 0.018 mm^2^.

**Figure 2:**
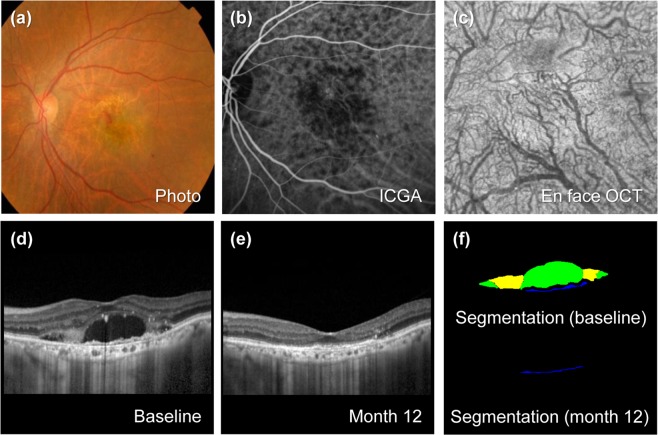
Representative case of typical neovascular age-related macular degeneration (nAMD) without pachychoroid. The patient is an 80-year-old woman with a baseline visual acuity of 20/100. For 45.4 months of follow-up, the eye was treated with 9 intravitreal injections of ranibizumab. (**a**) Baseline fundus photography shows subretinal exudation and hemorrhage change at macula. (**b**) Baseline indocyanine green angiography (ICGA) shows choroidal neovascularization (CNV) without polyps. (**c**) En face optical coherence tomography (En face OCT) scan at the final visit. Choroidal vessels spanning the macular area are not dilated. (**d**) Enhanced depth imaging optical coherence (EDI OCT) at baseline. The subfoveal choroidal thickness (SFCT) was thin as 91 µm. (**e**) EDI OCT scan 12 months from baseline. Visual acuity improved to 20/30 at 12 months. At the final visit, visual acuity improved to 20/40. (**f**) Upper panel shows predicted lesion corresponding baseline OCT B-scan by a convolutional neural network (CNN) (upper). Subretinal fluid (SRF, green color), subretinal hyperreflective material (SHRM, yellow color), and pigment epithelial detachment (PED, blue color) are noted. Each component could be quantified by converting the pixel counts into mm^2^, and the areas of SRF, SHRM and PED are 0.388, 0.143, and 0.027 mm^2^, respectively. Lower panel shows PED predicted from OCT B-scan at 12 months by CNN, and the area is 0.017 mm^2^.

**Figure 3:**
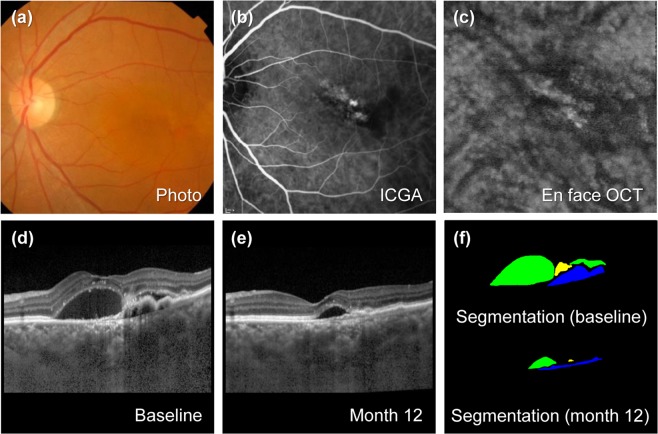
Representative ce of polypoidal choroidal vasculopathy (PCV) with pachychoroid. The patient is a 79-year-old woman with a baseline visual acuity of 20/40. For 31.0 months of follow-up, the eye was treated with intravitreal injections, including 14 aflibercept and 2 ranibizumab injections. (**a**) Baseline fundus photography shows subretinal fluid collection at macula. (**b**) Baseline indocyanine green angiography (ICGA) shows polypoidal lesion. (**c**) En face optical coherence tomography (En face OCT) scan at the final visit. Dilated choroidal vessels are prominent. (**d**) Enhanced depth imaging optical coherence (EDI OCT) at baseline. Choroidal layer is thickened with subfoveal choroidal thickness (SFCT) of 551 µm. (**e**) EDI OCT scan after 12 months from baseline. All areas of the lesions decreased, and visual acuity was maintained at 20/40. At the final visit, visual acuity improved to 20/32. (**f**) Upper panel shows predicted lesion corresponding baseline OCT B-scan by a convolutional neural network (CNN) (upper). Subretinal fluid (SRF, green color), subretinal hyperreflective material (SHRM, yellow color), and pigment epithelial detachment (PED, blue color) are noted. Each component could be quantified by converting the pixel counts into mm^2^, and the areas of SRF, SHRM, and PED are 0.650, 0.048, and 0.193 mm^2^, respectively. Lower panel shows predicted areas of SRF, SHRM and PED by the CNN as 0.091, 0.004, and 0.073 mm^2^, respectively.

**Figure 4:**
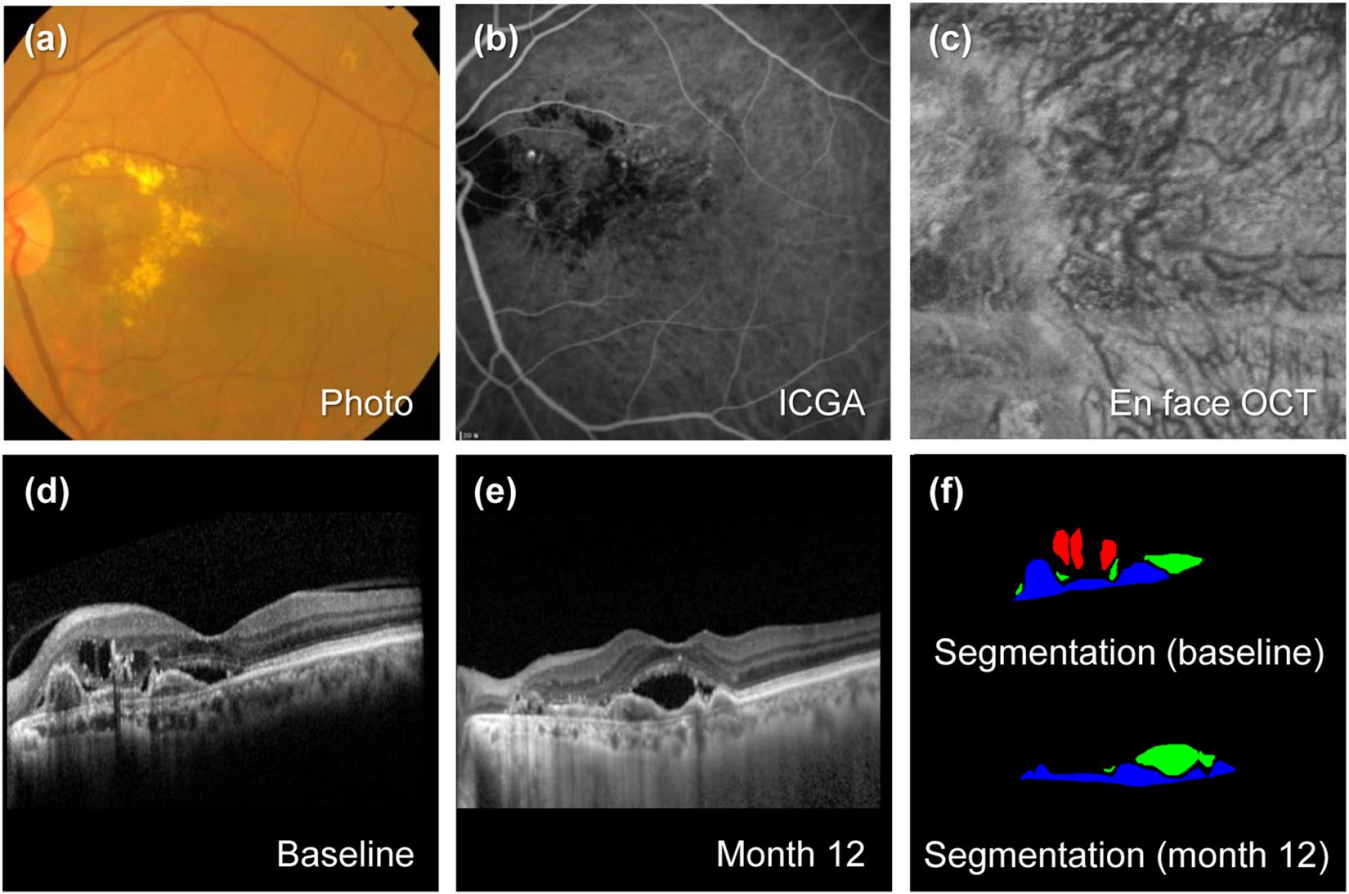
Representative case of polypoidal choroidal vasculopathy (PCV) without pachychoroid. The patient is a 78-year-old woman with a baseline visual acuity of 20/30. For 67.9 months of follow-up, the eye was treated with 10 intravitreal aflibercept injections. (**a**) Baseline fundus photography shows subretinal fluid, exudation with orange colored polyp at nasal side of fovea. (**b**) Baseline indocyanine green angiography (ICGA) shows peripapillary polypoidal change with branced vascular network. (**c**) En face optical coherence tomography (En face OCT) scan of the final visit. Choroidal vessels are not dilated. (**d**) Enhanced depth imaging optical coherence (EDI OCT) at baseline (lower). Choroidal layer is generally thin with a subfoveal choroidal thickness (SFCT) of 186 µm. (**e**) EDI OCT scan after 12 months from baseline. Intraretinal fluid (IRF) resolved, but subretinal fluid (SRF) and pigment epithelial detachment (PED) are still present, and visual acuity decreased to 20/50. Visual acuity at the final visit was 20/100. (**f**) Upper panel shows predicted lesion corresponding baseline OCT B-scan by a convolutional neural network (CNN). IRF (red color), SRF (green color), and PED (blue color) are noted. Each component could be quantified by converting the pixel counts into mm^2^, and the areas of IRF, SRF, and PED are 0.114, 0.090, and 0.236 mm^2^, respectively. Lower panel shows predicted areas of SRF and PED as 0.169 and 0.228 mm^2^, respectively.

Pachychoroid were present in 36 (27.3%) typical nAMD eyes and 19 (42.2%) PCV eyes. Detailed descriptions of the subgroups are depicted in Supplementary Table S2. The VA at any time point examined in typical nAMD with pachychoroid was better than that in typical nAMD without pachychoroid, while the VA between PCV with and without pachychoroid was not different. In typical nAMD, the number of eyes with VA gain among 36 eyes with pachychoroid was 17 (47.2%), while the number of eyes with VA gain among 96 eyes without pachychoroid was 25 (26.0%) (P = 0.03). By contrast, the number of PCV eyes with VA gain among 19 eyes of pachychoroid was 8 (42.1%), while the number of eyes with VA gain among 26 eyes without pachychoroid was 12 (46.2%), with no significant difference in the ratio (P = 0.79). Comparisons of the areas of 4 lesions between typical nAMD with and without pachychoroid revealed that the areas of IRF at baseline and month 12 were significantly smaller in typical nAMD with pachychoroid (Supplementary Table S2). By contrast, area of IRF was not different between PCV with and without pachychoroid at baseline and month 12 (Supplementary Table S2). Area of baseline SRF was larger in PCV with pachychoroid than that without pachychoroid (Supplementary Table S2). Representative images of typical nAMD and PCV with and without pachychoroid are depicted in Figs. 1–4.

### Factors associated with VA gain at final visit in typical nAMD and PCV

In univariate regression analyses, VA gain in typical nAMD was associated with poorer baseline VA and better VA at month 12, larger SHRM at baseline, greater decreases in SRF and SHRM, higher SFCT at baseline and the presence of pachychoroid (Supplementary Table S3). In multivariable regression analyses, VA gain in typical nAMD was associated with poorer baseline VA and better VA at month 12 (OR 88.938, CI 9.588–825.006, P < 0.001; OR 0.021, CI 0.002–0.199, P < 0.001, respectively). Considering SD OCT-associated factors only in multivariable regression analysis, VA gain in typical nAMD was associated with smaller baseline SHRM, greater decreases in SRF and SHRM during 12 months (OR 0.167, CI 0.034–0.822, P = 0.03; OR 1.564, CI 1.086–2.253, P = 0.02; OR 12.877, CI 2.079–79.763, P = 0.01, respectively) (Table 1). When the significance level was modified as 0.008 (0.05 divided by 6 factors, Table 1) by Bonferroni correction in the multivariable regression analysis, greater decreases in SHRM during 12 months was associated with VA gain.

**Table 1:** Multivariable logistic regression analysis for parameters associated with vision gain or initial logMAR 0.63 maintenance in typical age-related macular degeneration*.

Parameters	OR	95% CI	*P* Value
Baseline SHRM	0.167	0.034–0.822	0.028
Change amount of SRF	1.564	1.086–2.253	0.016
Change amount of SHRM	12.877	2.079–79.763	0.006
Age^†^	0.987	0.943–1.033	0.584
Gender^†^	1.021	0.428–2.440	0.962
Number of injections for first 12 months^†^	0.729	0.555–0.958	0.023

LogMAR = logarithm of the minimum angle of resolution; OR = odd ratio; CI = confidence interval; SRF = subretinal fluid; SHRM = subretinal hyperreflective material.

*Among the significant factors in univariate logistic regression analysis, factors associated with SD OCT, i.e., information about the area of IRF, SRF, PED, SHRM and the presence of pachychoroid, were considered for multivariable logistic regression analysis only. Units of lesion areas were converted to 0.1 mm^2^.

^†^Age, sex and the number of injections for first 12 months were adjusted.

In PCV, VA gain was associated with poorer baseline VA only in univariate analysis. No SD OCT-associated lesion components were associated with VA gain (Supplementary Table S4).

### Factors associated with VA gain at final visit in typical nAMD with and without pachychoroid

In univariate regression analyses, VA gain in typical nAMD with pachychoroid was associated with poorer VA at baseline only (Supplementary Table S5). Alternatively, VA gain in typical nAMD without pachychoroid was associated with poorer baseline VA, better VA at month 12, greater decreases in SRF and SHRM (Supplementary Table S6). In multivariable analysis, VA gain in typical nAMD without pachychoroid was associated with poorer baseline VA and better VA at month 12 (OR 58.801, CI 6.286–550.074, P < 0.001; OR 0.020, CI 0.002–0.186, P = 0.001, respectively). Considering SD OCT-associated factors only in multivariable regression analysis, VA gain in typical nAMD without pachychoroid was associated with greater decreases in SRF and SHRM during 12 months (OR 1.574, CI 1.042–2.378, P = 0.03; OR 1.775, CI 1.037–3.039. P = 0.04, respectively) (Table 2). However, when the significance level was modified as 0.01 (0.05 divided by 5 factors, Table 2) by Bonferroni correction, either factors was not found to be significant.

**Table 2:** Multivariable logistic regression analysis for parameters associated with vision gain or initial logmar 0.63 maintenance in typical age-related macular degeneration without pachychoroid*.

Parameters	OR	95% CI	*P* Value
Change amount of SRF	1.574	1.042–2.378	0.031
Change amount of SHRM	1.775	1.037–3.039	0.036
Age^†^	1.012	0.942–1.087	0.742
Gender^†^	1.194	0.437–3.261	0.729
Number of injections for first 12 months^†^	0.830	0.605–1.137	0.245

LogMAR = logarithm of the minimum angle of resolution; OR = odd ratio; CI = confidence interval; SRF = subretinal fluid; SHRM = subretinal hyperreflective material.

*Among the significant factors in univariate logistic regression analysis, factors associated with SD OCT, i.e., information about the area of IRF, SRF, PED, SHRM and the presence of pachychoroid, were considered for multivariable logistic regression analysis only. Units of lesion areas were converted to 0.1 mm^2^.

^†^Age, sex and the number of injections for first 12 months were adjusted.

### Factors associated with VA gain at final visit in PCV with and without pachychoroid

In univariate regression analyses, VA gain in PCV with pachychoroid was not associated with any factors (Supplementary Table S7). For PCV without pachychoroid, no factors were associated in univariate regression analysis (Supplementary Table S8).

## Discussion

To our best knowledge, this case series is the first to use a CNN to measure quantitative changes in retinal lesions on SD OCT in treatment-naïve CNV secondary to typical nAMD and PCV during 1 year of anti-VEGF treatments for the identification of imaging factors on SD OCT associated with the final visual outcome. Compared with measurements by clinicians, measurements by the CNN of the areas of IRF, SRF, PED and SHRM on SD OCT from 177 eyes were achieved in much less time. The presence of pachychoroid was also examined with the assumption that it could affect the visual outcome. Previous studies regarding the association between increased or decreased choroidal thickness and visual outcome in either AMD or PCV have shown controversial results^19–22^. The implication of pachychoroid combined with the quantitatively measured lesions in visual outcome are barely known.

When considering morphological factors only without regard to VA, factors associated with visual gain showed different patterns depending on the subgroups. In typical nAMD, baseline factors associated with VA gain were smaller SHRM at baseline, greater decreases in SRF and SHRM at month 12, while no morphological factors were associated in PCV. When the significance level was adjusted to 0.008 by Bonferroni correction, we could select the associated factor more conservatively, and the greater decrease in SHRM at month 12 was the factor associated with VA gain.

Interestingly, when typical nAMD and PCV were further subdivided based on the presence of pachychoroid, the association between VA gain and the reduction in SRF and SHRM at month 12 was significant only in typical nAMD without pachychoroid in multivariable regression analysis. However, the association became insignificant when Bonferroni correction was applied, although these factors were significant in the conventional significance level of 0.05. Regarding the impact of SRF on visual function, the presence of SRF at any time is associated with higher VA levels^10,20,21^, and the presence of SRF at baseline is a predictor of greater VA gain^20,22^. In the present study, the reduction in SRF was associated with VA gain in typical nAMD without pachychoroid; moreover, the positive effect of baseline SRF on visual outcome was found in typical nAMD without pachychoroid only. The amount of SRF in typical nAMD with pachychoroid or PCV might have a smaller effect on visual outcome than that in typical nAMD without pachychoroid. A similar trend was observed for the changes in SHRM. The beneficial effect of reduced SHRM on VA gain was found in typical nAMD without pachychoroid only. SHRM may have an adverse effect on VA in patients with AMD^11,23,24^. Our result showing that the significant association between visual gain and the reduction in SRF and SHRM in eyes with typical nAMD without pachychoroid only is intriguing because it is consistent with previous prospective studies that analyzed AMD eyes without considering the effect of choroidal morphological changes^22,25^. Although directly comparing previous results with our own results is difficult, we speculate that cohorts in previous studies predominantly comprised patients with typical nAMD without pachychoroid; therefore, SRF and SHRM emerged as the factors associated with the visual prognosis. However, a remaining question is why changes in SRF and SHRM were not related to VA gain in typical nAMD with pachychoroid or PCV. In other words, did vision improve regardless of changes in SRF or SHRM in these patients in the present study?

Compared with typical nAMD with pachychoroid, typical nAMD without pachychoroid maintained a larger IRF area from baseline to 12 months after treatment. IRF is a well-known predictive factor for poor visual outcome^8^. Therefore, the poor visual outcome in typical nAMD without pachychoroid might be due to the initial presence of greater IRF. Indeed, all VA-associated parameters of typical nAMD without pachychoroid, including baseline VA, VA after 12 months and VA at the final visit, were worse than those of typical nAMD with pachychoroid. Nevertheless, there were no differences in VA-associated parameters (baseline VA, VA at month 12, change in VA) and IRF area between PCV with pachychoroid and PCV without pachychoroid. Although why the absence of pachychoroid is associated with higher IRF in AMD patients is currently unclear, we speculate that among typical nAMD eyes without pachychoroid, only eyes with significant reduction in SRF and SHRM might gain vision despite the detrimental effect of IRF. Alternatively, in typical nAMD eyes with pachychoroid or all PCV eyes, IRF might be less developed, and these eyes might have a tendency to gain vision regardless of the amount of the reduction in SRF and SHRM. Furthermore, in these eyes, certain unknown choroidal factors in addition to the presence of pachychoroid are more relevant to visual outcomes than changes in pathologic retinal components, which might be negligible compared to more influential choroidal factors. Certain characteristics of the choroid, at least not the direct cause of typical nAMD or PCV, could be a factor influencing the outcome of the anti-VEGF treatment.

In addition to the influence of IRF in typical nAMD eyes without pachychoroid but not in typical nAMD with pachychoroid, we speculate that in thin choroid, choroidal vessels are packed in a small space; thus, the overlying choriocapillaris might have had a higher chance of compression by mechanical pressure. Conversely, in a normal or thick choroid, choroidal vessels would be surrounded by relatively more stromal space, and the overlying choriocapillaris might experience less pressure from the bulky choroidal vessels, circumventing the ischemic environment. Because we did not investigate the morphology of the choriocapillaris, further study is needed to analyze the association between pachychroroid and choriocapillaris morphology.

This study has several limitations. First, segmentation of lesion was only done on a single foveal OCT section and may not be reflective of the entire volume change of the lesion. Segmentation of lesions for the entire foveal scans might give volumetric and spatial information of lesions. Further study is needed to analyze the comprehensive anatomical information including three dimensional analysis of lesions combined with the choroidal thickness. Second, as described above, we investigated only the presence of pachychoroid without investigating changes in the overlying choriocapillaris. Third, the study design is retrospective; therefore, the treatment schedule and follow-up period were not well controlled. Furthermore, the number of injections is heterogeneous among groups. Finally, the number of eyes in each four subgroups is small, particularly in the nAMD with pachychoroid group and PCV groups, and this could lead to the absence of significant factors associated with visual acuity gain in these groups. Also, in the nAMD without pachychoroid group, visual gain at the final visit was associated with a greater decrease in SRF and SHRM in multivariate analysis (the significance level was set to 0.05). However, application of Bonferroni correction which selects the significant factors in multivariable regression analysis more conservatively, no factors remained associated in nAMD without pachychoroid group. Further study with larger number of patients might give more convincing results. However, the strengths of the present study included quantitative analysis of pathologic retinal components on SD OCT by the aid of a CNN from four classified groups of patients. The patients were divided into two groups, typical nAMD and PCV, and the analysis was further conducted on the subgroups according to the presence or absence of pachychoroid.

In conclusion, the reduction in SRF and SHRM area at 12 months was associated with good visual outcome in the typical nAMD without pachychoroid group but not in the other three groups. The current findings on the quantified area of pathologic lesions from automated segmentation aided by a CNN and the presence of pachychoroid on OCT can facilitate the identification of long-term visual outcomes-associated factors for CNV patients with various phenotypes during anti-VEGF treatments in more detail. Our discovery of the association between pathologic retinal components on SD OCT in typical nAMD and PCV patients suggests that SRF and SHRM may be clinically significant imaging biomarkers associated with the visual outcome and clinical course following anti-VEGF treatments, particularly in typical nAMD patients without pachychoroid. Additionally, in typical AMD eyes with pachychoroid or PCV eyes, visual prognosis could be favorable regardless of changes in SRF or SHRM during anti-VEGF treatments.

## Methods

### Participants

This study was performed according to the tenets of the Declaration of Helsinki and was approved by the institutional review board of Konkuk University Medical Center (KUH 1100069), which allowed exemption from the written informed consent because of the study’s retrospective design. We retrospectively reviewed the medical records of 273 patients (aged 50 years or older) with typical nAMD or PCV who visited Konkuk University Medical Center from January 2010 through September 2018 and had no history of prior treatment for nAMD or PCV or intraocular surgery except cataract operation. In this study, typical nAMD was defined as nAMD containing any type of CNV (type 1, 2, and 3) confirmed by multimodal retinal imaging (FA, indocyanine green angiography [ICGA], and SD OCT). The image resolution of OCT image was 512 pixels by 496 pixels for width and height, and the actual length was 5.8 by 1.9 mm, respectively. The diagnosis of PCV was based on ICGA findings of polypoidal lesions. SFCT was measured manually using the caliper function in EDI OCT (Spectralis OCT2, Heidelberg Engineering, Heidelberg, Germany). Eyes with the presence of pachychoroid were defined as those with SFCT ≥ 300 μm, with prominently dilated outer choroidal vessels (pachyvessels) observed in at least one out of 5 horizontal B-scans of EDI OCT, which are 250 µm apart. Two graders (H.L and H.C) independently assessed the EDI OCT scans. For this study, out of these 283 eyes of 273 treatment-naïve patients, we analyzed 177 eyes of 169 patients. A total of 106 eyes were excluded for the following reasons: 66 eyes with no medical record after 12 months due to loss during follow-up or skipping the anticipated follow-up date, 4 eyes with baseline best corrected VA (BCVA) less than 20/320 Snellen equivalent, 30 eyes with poor image quality or a large fibrotic scar in the macula that prevented proper segmentation of lesions, 4 eyes with high myopia over -6 diopters, and 2 eyes with advanced glaucoma. No eyes with chorioretinal atrophic changes secondary to pathologic myopia or coexisting macular diseases that may influence VA were included.

In addition, eyes with pachychoroid neovascularization (PNV) were included in the group of typical nAMD with pachychoroid. Although the criteria for PNV are incompletely defined^26^, in this study, PNV was defined as choroidal neovascularization (CNV) accompanied by subfoveal choroidal thickness (SFCT) ≥ 300 μm with following conditions; no visible polyp on ICGA, no previous history of central serous chorioretinopathy and no drusen or drusen-like deposits in wide-field fundus photography (Optomap, Optos PLC. Dunfermline, UK). At baseline, all eyes underwent comprehensive ophthalmic examinations, including decimal BCVA, slit-lamp biomicroscopy, color fundus photography, fluorescein angiography (FA) and ICGA using confocal scanning laser ophthalmoscopy (Spectralis HRA + OCT; Heidelberg Engineering, Heidelberg, Germany), SD OCT volume scans and additional EDI scans for better resolution of the choroidal layer. At 12 months after the initial anti-VEGF treatment and at the final visit, all patients underwent the BCVA test, slit-lamp biomicroscopy, color fundus photography, SD OCT and additional EDI OCT.

All 177 eyes were treated with 3-month intravitreal anti-VEGF injections (either ranibizumab or aflibercept) that were maintained by a pro re nata regimen. According to the VA at the final visit, patients were grouped into the following categories: VA gainer and non-VA gainer groups. The VA gainer group was defined as patients who gained 0.3 or more logarithm of the minimum angle of resolution (logMAR) VA at final visit or whose baseline logMAR VA was 0.2 or better (Snellen equivalent 20/32 or better) and retained their VA throughout the follow-up period. The non-VA gainer group was defined as other patients not included in the VA gainer group.

### Automated segmentation and quantification of lesions in OCT

A pretrained deep neural network based on a CNN was used to segment the IRF, SRF, PED, and SHRM on SD OCT images. Briefly, this network was trained by 11,550 augmented images from 550 B-scans. Detailed information regarding this network is described in our previous study^18^. An image dataset containing 354 horizontal OCT B-scans (177 baseline scans and 177 month 12 scans spanning the foveal center) was entered into the CNN, and the segmented lesions were inspected by a senior clinician (H.C). When segmentation errors were detected, they were adjusted by ImageJ by a clinician (H.L and M.C) and reevaluated by the senior clinician (H.C). The pixels of the segmented area were counted using Python (ver. 3.6) and converted to the mm^2^ scale.

### Statistical analysis

Statistical analyses were performed using SPSS statistical software (version 19.0; SPSS Inc). BCVA was converted to logMAR for analysis. Comparisons of means between groups were made using the Mann-Whitney test. To identify factors affecting VA gain, we performed univariate logistic regression analysis, followed by stepwise multivariable logistic regression. All regression analyses were adjusted by age, sex and the number of injections in first 12 months. A P value less than 0.05 was considered statistically significant. Bonferroni correction was applied to the result from multivariable logistic regression analyses.

## Supplementary information

Supplementary information

## Data Availability

The datasets generated during and/or analyzed during the current study are not publicly available due to our hospital’s policy about patient records but are available from the corresponding author on reasonable request.
