# Supplementary material for: Association of imaging factors derived from convolutional neural network with visual outcomes in age-related macular degeneration and polypoidal choroidal vasculopathy
Source: Sci Rep. 2019 Dec 27;9:19857. doi: 10.1038/s41598-019-56420-z (PMC6934705; doi:10.1038/s41598-019-56420-z)
Supplement: Supplementary file 1 — Supplementary information [file 41598_2019_56420_MOESM1_ESM.pdf]

Association of imaging factors derived from convolutional neural network with visual outcomes in age-related macular degeneration and polypoidal choroidal vasculopathy

Hyungwoo Lee, MD, PhD[1]; Minsu Jang, MD[1]; Hyung Chan Kim, MD, PhD[1]; Hyewon Chung, MD, PhD[1]

Department of Ophthalmology, Konkuk University School of Medicine, Konkuk University Medical Center, Seoul, Republic of Korea[1]

Supplementary Table S1. Baseline study population and characteristics*

Parameters	Typical nAMD	PCV	*P* Value
Number of eyes, No.	132	45	
Age, yr	69.8 ± 9.0	65.0 ± 6.7	<0.001
Gender (M/F), No. (%)	74 (56.1%)/58 (43.9%)	33 (73.3%)/12 (26.7%)	0.04†
Total f/u, months	36.1 ± 24.7	43.6 ± 32.1	0.36
Injection for 12 months, No.	4.9 ± 1.7	5.3 ± 1.8	0.25
Total injection, No.	9.0 ± 6.5	11.9 ± 8.0	0.03
Visual acuity (logMAR)			
Baseline	0.507 ± 0.420	0.551 ± 0.523	0.91
After 3 loading doses	0.435 ± 0.395	0.378 ± 0.392	0.32
At 12 months	0.446 ± 0.422	0.395 ± 0.411	0.36
At final visit	0.656 ± 0.632	0.593 ± 0.634	0.42
SFCT, μm	233.0 ± 107.9	308.0 ± 104.0	<0.001
Autosegmented area on SD OCT, mm^2			
Baseline			
IRF	0.024 ± 0.060	0.011 ± 0.044	0.11
SRF	0.072 ± 0.125	0.209 ± 0.269	<0.001
SHRM	0.063 ± 0.108	0.090 ± 0.125	0.08
PED	0.125 ± 0.282	0.227 ± 0.313	0.001
At 12 months			
IRF	0.006 ± 0.023	0.011 ± 0.049	0.97
SRF	0.031 ± 0.091	0.044 ± 0.097	0.45
SHRM	0.032 ± 0.082	0.010 ± 0.031	0.16
PED	0.083 ± 0.167	0.090 ± 0.115	0.21
Change from baseline to 12 months			
IRF	0.018 ± 0.058	0.000 ± 0.015	0.02
SRF	0.041 ± 0.140	0.166 ± 0.272	0.001
SHRM	0.031 ± 0.117	0.080 ± 0.125	0.03
PED	0.042 ± 0.260	0.138 ± 0.285	0.04

Typical nAMD = typical neovascular age-related macular degeneration; PCV = polypoidal choroidal vasculopathy; M/F = male/female; f/u = follow-up period; logMAR = logarithm of the minimum angle of resolution; SFCT = subfoveal choroidal thickness (μm); SD OCT = spectral domain optical coherence tomography; IRF = intraretinal fluid; SRF = subretinal fluid; SHRM = subretinal hyperreflective material; PED = pigment epithelial detachment

* Values are shown as mean ± standard deviation, and the difference of mean between two groups was evaluated by the Mann-Whitney U test except for the gender ratio.

† Difference in the gender ratio was calculated using the Chi-square test.

Supplementary Table S2. Baseline study population and characteristics based on the presence and absence of pachychoroid*

	Typical nAMD			PCV		
**Parameters**	**Pachychoroid (+)**	**Pachychoroid (-)**	***P* Value**	**Pachychoroid (+)**	**Pachychoroid (-)**	***P* Value**
Number of eyes, No.	36	96		19	26	
Age, yr	64.1 ± 10.7	71.9 ± 7.3	<0.001	66.2 ± 7.6	64.2 (5.9)	0.42
Gender (M/F), No. (%)	26(72.2)/10(27.8)	48(50.0)/48(50.0)	0.02†	21(72.4)/8(27.6)	11(73.3)/4(26.7)	0.96†
Total f/u, months	35.3 ± 27.5	36.4 ± 23.7	0.38	43.7 ± 36.2	43.4 ± 29.5	0.68
Injection for 12 months, No.	4.6 ± 1.4	5.1 ± 1.7	0.17	5.3 ± 1.5	5.2 ± 2.0	0.76
Total injection, No.	8.5 ± 7.6	9.2 ± 6.1	0.14	11.6 ± 6.8	12.1 ± 8.9	0.87
Visual acuity (logMAR)						
Baseline	0.368 ± 0.396	0.559 ± 0.418	0.004	0.571 ± 0.578	0.537 ± 0.491	0.97
After 3 loading doses	0.278 ± 0.332	0.494 ± 0.402	0.001	0.286 ± 0.350	0.445 ± 0.413	0.13
At 12 months	0.256 ± 0.273	0.517 ± 0.446	0.001	0.314 ± 0.370	0.455 ± 0.436	0.23
At final visit	0.334 ± 0.454	0.776 ± 0.648	<0.001	0.738 ± 0.775	0.488 ± 0.497	0.62
SFCT, μm	378.4 ± 56.6	178.5 ± 62.3	<0.001	396.6 ± 98.0	243.3 ± 42.2	<0.001
Autosegmented area on SD OCT, mm^2						
Baseline						
IRF	0.008 ± 0.031	0.030 ± 0.067	0.01	0.013 ± 0.055	0.010 ± 0.035	0.67
SRF	0.072 ± 0.096	0.072 ± 0.135	0.45	0.244 ± 0.183	0.184 ± 0.319	0.04
SHRM	0.063 ± 0.106	0.063 ± 0.109	0.80	0.063 ± 0.115	0.110 ± 0.130	0.59
PED	0.148 ± 0.356	0.116 ± 0.250	0.66	0.160 ± 0.172	0.277 ± 0.381	0.75
At 12 months						
IRF	0.000 ± 0.000	0.008 ± 0.026	0.01	0.017 ± 0.064	0.007 ± 0.034	0.98
SRF	0.017 ± 0.048	0.036 ± 0.102	0.42	0.054 ± 0.120	0.036 ± 0.077	0.55
SHRM	0.015 ± 0.047	0.038 ± 0.092	0.19	0.009 ± 0.024	0.011 ± 0.035	0.52
PED	0.075 ± 0.131	0.086 ± 0.179	0.34	0.106 ± 0.143	0.077 ± 0.091	0.43
Change from baseline to 12 months						
IRF	0.008 ± 0.031	0.021 ± 0.065	0.20	-0.004 ± 0.014	0.003 ± 0.015	0.59
SRF	0.055 ± 0.102	0.036 ± 0.152	0.17	0.190 ± 0.202	0.148 ± 0.316	0.15
SHRM	0.048 ± 0.108	0.024 ± 0.120	0.55	0.054 ± 0.112	0.099 ± 0.132	0.94
PED	0.073 ± 0.261	0.030 ± 0.260	0.05	0.053 ± 0.177	0.199 ± 0.334	0.15

Typical nAMD = typical neovascular age-related macular degeneration; PCV = polypoidal choroidal vasculopathy; M/F = male/female; f/u = follow-up period; logMAR = logarithm of the minimum angle of resolution; SFCT = subfoveal choroidal thickness (μm); SD OCT = spectral domain optical coherence tomography; IRF = intraretinal fluid; SRF = subretinal fluid; SHRM = subretinal hyperreflective material; PED = pigment epithelial detachment

* Values are shown as mean ± standard deviation, and the difference of mean between two groups was evaluated by the Mann-Whitney U test except for the gender ratio.

† Difference in the gender ratio was calculated using the Chi-square test.

Supplementary Table S3. Univariable logistic regression analysis for parameters associated with vision gain or initial logmar 0.63 maintenance in typical age-related macular degeneration*

	Typical nAMD (n=132)				
**Parameters**	**OR**	**95% CI**			***P* Value**
Visual acuity (logMAR)					
Baseline	4.840	1.832	-	12.783	0.00
After 3 loading doses	0.588	0.201	-	1.723	0.33
At 12 months	0.174	0.050	-	0.606	0.01
SFCT	1.005	1.001	-	1.009	0.01
Autosegmented area on SD OCT					
Baseline					
IRF	0.746	0.354	-	1.572	0.44
SRF	1.325	0.978	-	1.796	0.07
SHRM	1.450	1.015	-	2.070	0.04
PED	0.937	0.795	-	1.104	0.44
At 12 months					
IRF	0.638	0.096	-	4.242	0.64
SRF	0.534	0.226	-	1.261	0.15
SHRM	0.318	0.098	-	1.035	0.06
PED	0.978	0.754	-	1.268	0.86
Change from baseline to 12 months					
IRF	0.798	0.387	-	1.647	0.54
SRF	1.538	1.091	-	2.170	0.01
SHRM	1.926	1.278	-	2.901	0.00
PED	0.934	0.787	-	1.108	0.43

Typical nAMD = typical neovascular age-related macular degeneration; OR = odd ratio, CI = confidence interval; logMAR = logarithm of the minimum angle of resolution; SFCT = subfoveal choroidal thickness (μm); SD OCT = spectral domain optical coherence tomography; IRF = intraretinal fluid; SRF = subretinal fluid; SHRM = subretinal hyperreflective material; PED = pigment epithelial detachment. Units of lesion areas were converted to 0.1 mm^2.

* Age, sex and the number of injections for first 12 months were adjusted.

Supplementary Table S4. Univariable logistic regression analysis for parameters associated with vision gain or initial logmar 0.63 maintenance in polypoidal choroidal vasculopathy*

Parameters	PCV (n=45)				
	OR	95% CI			*P* Value
Visual acuity (logMAR)					
Baseline	10.187	1.704	-	60.907	0.01
After 3 loading doses	2.919	0.507	-	16.813	0.23
At 12 months	0.620	0.118	-	3.268	0.57
SFCT	0.997	0.991	-	1.004	0.46
Autosegmented area on SD OCT					
Baseline					
IRF	9.728	0.247	-	382.930	0.22
SRF	1.144	0.880	-	1.486	0.31
SHRM	1.325	0.788	-	2.226	0.29
PED	1.117	0.916	-	1.362	0.28
At 12 months					
IRF	8.464	0.113	-	633.039	0.33
SRF	0.732	0.351	-	1.523	0.40
SHRM	0.842	0.062	-	11.341	0.90
PED	0.584	0.265	-	1.287	0.18
Change from baseline to 12 months					
IRF	0.487	0.003	-	69.604	0.78
SRF	1.204	0.905	-	1.602	0.20
SHRM	1.345	0.791	-	2.287	0.27
PED	1.216	0.959	-	1.543	0.11

PCV = polypoidal choroidal vasculopathy; OR = odd ratio, CI = confidence interval; logMAR = logarithm of the minimum angle of resolution; SFCT = subfoveal choroidal thickness (μm); SD OCT = spectral domain optical coherence tomography; IRF = intraretinal fluid; SRF = subretinal fluid; SHRM = subretinal hyperreflective material; PED = pigment epithelial detachment. Units of lesion areas were converted to 0.1 mm^2.

* Age, sex and the number of injections for first 12 months were adjusted.

Supplementary Table S5. Univariable logistic regression analysis for parameters associated with vision gain or initial logmar 0.63 maintenance in typical age-related macular degeneration with pachychoroid*

	Typical nAMD with pachychoroid (n=36)				
**Parameters**	**OR**	**95% CI**			***P* Value**
Visual acuity (logMAR)					
Baseline	9.628	0.811	-	114.372	0.02
After 3 loading doses	1.522	0.163	-	14.216	0.60
At 12 months	0.568	0.033	-	9.828	0.25
SFCT	1.007	0.994	-	1.020	0.31
Autosegmented area on SD OCT					
Baseline					
IRF	0.529	0.052	-	5.417	0.59
SRF	1.093	0.525	-	2.275	0.81
SHRM	1.442	0.693	-	2.998	0.33
PED	0.734	0.405	-	1.330	0.31
At 12 months					
IRF	N/A		N/A		N/A
SRF	0.245	0.026	-	2.302	0.22
SHRM	0.353	0.022	-	5.766	0.46
PED	0.553	0.216	-	1.418	0.22
Change from baseline to 12 months					
IRF	0.529	0.052	-	5.417	0.59
SRF	1.423	0.654	-	3.098	0.37
SHRM	1.657	0.773	-	3.553	0.19
PED	0.806	0.500	-	1.301	0.38

Typical nAMD = typical neovascular age-related macular degeneration; OR = odd ratio, CI = confidence interval; logMAR = logarithm of the minimum angle of resolution; SFCT = subfoveal choroidal thickness (μm); SD OCT = spectral domain optical coherence tomography; IRF = intraretinal fluid; SRF = subretinal fluid; SHRM = subretinal hyperreflective material; PED = pigment epithelial detachment; N/A = Not applicable. Units of lesion areas were converted to 0.1 mm^2.

* Age, sex and the number of injections for first 12 months were adjusted.

Supplementary Table S6. Univariable logistic regression analysis for parameters associated with vision gain or initial logmar 0.63 maintenance in typical age-related macular degeneration without pachychoroid *

	**Typical nAMD without pachychoroid (n=96)**				
**Parameters**	**OR**	**95% CI**			***P* Value**
Visual acuity (logMAR)					
Baseline	5.793	1.743	-	19.253	0.00
After 3 loading doses	0.585	0.161	-	2.122	0.41
At 12 months	0.174	0.042	-	0.718	0.02
SFCT	1.005	0.997	-	1.013	0.21
Autosegmented area on SD OCT					
Baseline					
IRF	0.832	0.381	-	1.815	0.64
SRF	1.407	1.001	-	1.978	0.05
SHRM	1.295	0.840	-	1.998	0.24
PED	0.990	0.819	-	1.195	0.91
At 12 months					
IRF	0.703	0.102	-	4.839	0.72
SRF	0.642	0.266	-	1.548	0.32
SHRM	0.334	0.090	-	1.241	0.10
PED	1.034	0.795	-	1.346	0.80
Change from baseline to 12 months					
IRF	0.880	0.412	-	1.883	0.74
SRF	1.588	1.070	-	2.356	0.02
SHRM	1.806	1.087	-	3.002	0.02
PED	0.972	0.797	-	1.187	0.78

Typical nAMD = typical neovascular age-related macular degeneration; OR = odd ratio, CI = confidence interval; logMAR = logarithm of the minimum angle of resolution; SFCT = subfoveal choroidal thickness (μm); SD OCT = spectral domain optical coherence tomography; IRF = intraretinal fluid; SRF = subretinal fluid; SHRM = subretinal hyperreflective material; PED = pigment epithelial detachment. Units of lesion areas were converted to 0.1 mm^2.

* Age, sex and the number of injections for first 12 months were adjusted.

Supplementary Table S7. Univariable logistic regression analysis for parameters associated with vision gain or initial logmar 0.63 maintenance in polypoidal choroidal vasculopathy with pachychoroid*

	PCV with pachychoroid (n=19)				
**Parameters**	**OR**	**95% CI**			**P value**
Visual acuity (logMAR)					
Baseline	145.685	0.345	-	6.147×10^{5}	0.11
After 3 loading doses	17.716	0.129	-	2438.725	0.25
At 12 months	3.552	0.049	-	258.305	0.56
SFCT	0.992	0.980	-	1.005	0.25
Autosegmented area on SD OCT					
Baseline					
IRF	>999.999	<0.001	-	>999.999	1.00
SRF	0.203	0.022	-	1.908	0.16
SHRM	0.971	0.347	-	2.718	0.96
PED	2.503	0.826	-	7.592	0.10
At 12 months					
IRF	18.829	<0.001	-	6.317×10^{6}	0.65
SRF	0.695	0.253	-	1.910	0.48
SHRM	1.125×10^{6}	<0.001	-	1.619×10^{16}	0.24
PED	1.455	0.512	-	4.137	0.48
Change from baseline to 12 months					
IRF	<0.001	<0.001	-	143.385	0.20
SRF	0.273	0.036	-	2.070	0.21
SHRM	0.763	0.256	-	2.270	0.63
PED	1.726	0.683	-	4.364	0.25

PCV = polypoidal choroidal vasculopathy; OR = odd ratio, CI = confidence interval; logMAR = logarithm of the minimum angle of resolution; SFCT = subfoveal choroidal thickness (μm); SD OCT = spectral domain optical coherence tomography; IRF = intraretinal fluid; SRF = subretinal fluid; SHRM = subretinal hyperreflective material; PED = pigment epithelial detachment. Units of lesion areas were converted to 0.1 mm^2.

* Age, sex and the number of injections were adjusted.

Supplementary Table S8. Univariable logistic regression analysis for parameters associated with vision gain or initial logmar 0.63 maintenance in polypoidal choroidal vasculopathy without pachychoroid*

	PCV without pachychoroid (n=26)				
**Parameters**	**OR**	**95% CI**			**P value**
Visual acuity (logMAR)					
Baseline	6.965	0.825	-	58.787	0.07
After 3 loading doses	4.025	0.411	-	39.423	0.23
At 12 months	0.572	0.073	-	4.478	0.59
SFCT	1.002	0.980	-	1.025	0.85
Autosegmented area on SD OCT					
Baseline					
IRF	4.060	0.144	-	114.146	0.41
SRF	1.590	0.764	-	3.309	0.21
SHRM	1.363	0.691	-	2.689	0.37
PED	1.122	0.898	-	1.403	0.31
At 12 months					
IRF	25.708	<0.001	-	3.092×10^8	0.70
SRF	0.346	0.048	-	2.484	0.29
SHRM	<0.001	<0.001	-	202.280	0.20
PED	0.256	0.065	-	1.018	0.05
Change from baseline to 12 months					
IRF	0.284	<0.001	-	194.748	0.71
SRF	1.772	0.865	-	3.632	0.12
SHRM	1.518	0.743	-	3.100	0.25
PED	1.252	0.953	-	1.646	0.11

PCV = polypoidal choroidal vasculopathy; OR = odd ratio, CI = confidence interval; logMAR = logarithm of the minimum angle of resolution; SFCT = subfoveal choroidal thickness (μm); SD OCT = spectral domain optical coherence tomography; IRF = intraretinal fluid; SRF = subretinal fluid; SHRM = subretinal hyperreflective material; PED = pigment epithelial detachment. Units of lesion areas were converted to 0.1 mm^2.

* Age, sex and the number of injections were adjusted.
